# Submicroscopic malaria infection is not associated with fever in cross-sectional studies in Malawi

**DOI:** 10.1186/s12936-020-03296-4

**Published:** 2020-06-29

**Authors:** Jimmy Vareta, Andrea G. Buchwald, Angelica Barrall, Lauren M. Cohee, Jenny A. Walldorf, Jenna E. Coalson, Karl Seydel, Alick Sixpence, Don P. Mathanga, Terrie E. Taylor, Miriam K. Laufer

**Affiliations:** 1 Center for Vaccine Development and Global Health, University of Maryland School of Medicine, Baltimore, MD USA; 2 School of Public Health, University of Maryland College Park, College Park, MD USA; 3 Department of Epidemiology, University of Michigan School of Public Health, Ann Arbor, MI USA; 4 Department of Osteopathic Medical Specialties, College of Osteopathic Medicine, Michigan State University, East Lansing, MI USA; 5 Malaria Alert Center, College of Medicine, University of Malawi, Blantyre, Malawi; 6 Present Address: Colorado School of Public Health, University of Colorado, Denver, USA; 7 Present Address: Fielding School of Public Health, University of California, Los Angeles, USA; 8 Present Address: US Center for Disease Control and Prevention, Atlanta, GA USA; 9 Present Address: University of Notre Dame, Notre Dame, IN USA

**Keywords:** Malaria, Submicroscopic infection, *Plasmodium falciparum*

## Abstract

**Background:**

Submicroscopic *Plasmodium falciparum* infections are widespread in many areas. However, the contribution of these infections to symptomatic malaria is not well understood. This study evaluated whether participants with submicroscopic *P*. *falciparum* infections have higher prevalence of fever than uninfected participants in southern Malawi.

**Methods:**

A total of 16,650 children and adults were enrolled in the course of six cross-sectional surveys during the dry season (October–November) and after the rainy season (April–May) between 2012 and 2014 in three districts in southern Malawi. Demographic and socioeconomic data were collected in conjunction with data on clinical histories, use of malaria preventive measures, and anti-malarial medication taken within 2 weeks of the survey. Axillary temperatures were measured, and blood samples were collected for *P. falciparum* detection by microscopy and PCR. Participants without malaria parasites detected on microscopy but with a positive PCR for *P. falciparum* were defined as having submicroscopic infection. Fever was defined as having any one of: reported fever in the past 2 weeks, reported fever in the past 48 h, or a temperature of ≥ 37.5 °C measured at the time of interview.

**Results:**

Submicroscopic *P. falciparum* infections and fever were both detected in 9% of the study population. In the final analysis adjusted for clustering within household and enumeration area, having submicroscopic *P. falciparum* infection was associated with reduced odds of fever in the dry season (odds ratio = 0.52; 95% CI 0.33–0.82); the association in the rainy season did not achieve statistical significance (odds ratio = 1.20; 95% CI 0.91–1.59). The association between submicroscopic infection and fever was consistent across all age groups. When the definition of fever was limited to temperature of ≥ 37.5 °C measured at the time of interview, the association was not statistically significant in either the rainy or dry season.

**Conclusions:**

In this series of cross-sectional studies in southern Malawi, submicroscopic *P. falciparum* infection was not associated with increased risk of fever. Submicroscopic detection of the malaria parasite is important in efforts to decrease transmission but is not essential for the clinical recognition of malaria disease.

## Background

Despite major public health efforts to eliminate malaria worldwide, the incidence of malaria illnesses is still high in many areas, particularly in sub-Saharan Africa [1]. Although prompt recognition and treatment of malaria illnesses with artemisinin-based combination therapy is increasing, and measures to reduce the transmission of malaria parasites (e.g., indoor residual spraying and long-lasting insecticide-treated bed nets) are being widely used in many malaria-endemic areas [2, 3], there is growing evidence that low-density *Plasmodium falciparum* infections remain widespread [4–8]. These low-density *P. falciparum* infections act as transmission reservoir of malaria [9–11], however, their contribution to symptomatic malaria is not well understood.

Accurate estimation of the incidence and prevalence of *P. falciparum* infections in many geographical areas has been difficult because microscopy, the gold standard for the clinical diagnosis of malaria illnesses, and rapid diagnostic tests (RDTs) are not sensitive enough to detect low-density parasitaemia [12, 13]. The increasing use of molecular methods in many research settings has uncovered a large reservoir of submicroscopic *P. falciparum* infections [14, 15]. However, molecular methods are expensive and would be difficult to implement for routine diagnosis of clinical episodes of malaria in resource-limited settings without concrete evidence showing that these infections contribute to malaria illnesses. The goal of this study was to elucidate the clinical impact of submicroscopic malaria infections detected only by sensitive molecular techniques such as PCR [16, 17].

Others have evaluated the association between submicroscopic *P. falciparum* infections and clinical malaria [12, 18–21], but with varying study designs and inconsistent results. Most recently, a study from Uganda suggested that submicroscopic *P. falciparum* infections may be associated with symptoms [19]. This report and other studies have focused on young children who have not developed the acquired immunity to clinical malaria that is typical of older children and adults in high transmission settings. A large data set from six household-based cross-sectional surveys that were conducted in three districts in southern Malawi between 2012 and 2014 was analyzed to assess the association between submicroscopic *P. falciparum* infection and fever in different transmission settings and seasons, and across all age groups. This study tested the hypothesis that submicroscopic *P. falciparum* infection would be positively associated with fever and that the association would differ by season, transmission intensity and age. These are all factors that affect force of infection and acquired immunity that would modulate the likelihood of fever in the presence of low-level parasitaemia.

## Methods

### Study design and setting

Six household-based cross-sectional surveys were conducted in three districts in southern Malawi between 2012 and 2014 to characterize the burden of *P. falciparum* infection and to understand factors that influence parasite carriage. The three districts were: Blantyre, an urban and peri-urban highland with low transmission; Chikhwawa, a lowland with high transmission; and Thyolo, a highland with moderate transmission. The surveys were carried out twice a year for a period of 6 weeks: soon after the rainy season (April and May) and in the dry season (October and November).

### Study population and sampling methods

The surveys included all individuals over six months of age from households that were randomly selected in Blantyre, Chikhwawa and Thyolo districts. Briefly, 10 enumeration areas (EA) were randomly selected from each district. The 10 EAs per district were divided into compact segments containing 30 households each. One segment was selected from each EA to be included in the study and the same compact segments were visited for each survey. All households in the segment selected were eligible for the study; however, if one of the households did not meet the criteria for inclusion in the study it was replaced by the nearest household [17]. Same houses were sampled at each visit (survey), but information to aid in tracking survey participants to determine if they had repeated observations was not collected.

The surveys targeted all members of the household who had slept in the house for at least 2 weeks of the month prior to the survey. The analysis included household members aged 6 months old and above. Household visitors and individuals from households where the eldest person present at the time of the survey was a minor were excluded from the survey. Consent to participate in the study was sought from the head of the household and from each adult present. Parents or guardians were asked to provide consent on behalf of the children below the age of 18 years old and assent was sought from children who were 13–17 years old. Once consent and assent were granted, interviewers collected data on demographic and socioeconomic status (SES) variables, malaria preventive measures used, and any anti-malarial medication taken within 2 weeks of the survey. In addition, axillary temperature was measured using digital thermometers, and peripheral blood samples were collected on glass slides (thick and thin films) for malaria microscopy and on Whatman 3MM filter paper (GE Healthcare Ltd., New Jersey, USA) for molecular testing as described previously [22].

### Definitions

The primary outcome of interest was fever. Three fever measures were collected: reported fever in the past two weeks, reported fever in the past 48 h and temperature ≥ 37.5 °C measured at the time of specimen collection. In this study, for the primary analysis, fever was defined as having a fever using at least one of the three fever measures outlined above. A secondary analysis was conducted with objective fever as the outcome, and this was defined as axillary temperature of ≥ 37.5 °C. Submicroscopic *P. falciparum* infection was defined as a positive result by real-time PCR targeting *P. falciparum* lactate dehydrogenase gene and a negative malaria microscopy result. Details of the procedures for *P. falciparum* detection using real-time PCR and microscopy have been previously described [17, 23]. Individuals who tested negative by both microscopy and PCR were included as the comparison group. Participant ages were categorized as follows: 6 months–5 years, 6–15 year and 16 years and above. For SES, ten indicator variables were used to derive a wealth index weighted based on principal component analysis methods as described by Filmer and Pritchett [24]. The survey households were then subdivided into four wealth quartiles based on the weighted indicator score.

### Data analysis

Median age in years and proportion of individuals were estimated within strata of sex, SES quartile, season and age group in each district. Proportion of individuals with and without submicroscopic *P. falciparum* infection and proportion of individuals with and without fever were computed within strata of district, season, and age groups. Mixed effects logistic regression models were used to estimate the association between submicroscopic *P. falciparum* infection and fever within strata age groups, districts, SES quartile and season, while accounting for clustering within household and EA. In the final model comparing the log odds of fever between submicroscopic *P. falciparum* infection and no parasitaemia, district, participant age and season were included as covariates. In addition, a term for the interaction between submicroscopic infection and season (submicroscopic infection*season) was included in the final model. To determine if the association between submicroscopic infection and fever would be different when using broad (at least one of these three measures: reported fever in the past 2 weeks, reported fever in the past 48 h and temperature ≥ 37.5 °C measured at the time of the interview and specimen) or narrow (objective fever) definition of fever, we conducted a sensitivity analysis using objective fever (axillary temperature of ≥ 37.5 °C measured at the time of the interview) as the outcome of interest. All data analyses were done using SAS 9.4 (SAS Institute Inc., Cary, North Carolina) statistical software and were conducted at alpha level of 0.05.

## Results

### Survey population characteristics

A total of 16,650 people participated in the six surveys conducted between 2012 and 2014 in Blantyre, Chikhwawa and Thyolo districts. The median age of survey participants was 13 (IQR: 6–27) in Blantyre, 12 in Chikhwawa (IQR: 6–28), and 13 in Thyolo (IQR: 6–30). The distribution of participants within sex, age groups, and seasonal categories was similar in the three districts (Table 1). Blantyre residents tended to have higher scores on the asset-based SES index. More of the participants in Chikhwawa and Thyolo (34.5% and 31.9% respectively) were in the lowest SES quartile compared to Blantyre (3.3%). In Blantyre District, 59.1% of study participants were in the highest SES quartile compared to only 7.9% in Chikhwawa and 13.3% in Thyolo. The characteristics of the study populations grouped by district are described in Table 1.

**Table 1:** Characteristics of the survey population in three districts

Characteristic	Number of Participants (Column %)	Blantyre	Chikhwawa	Thyolo
Number	16650	5333	5830	5487
Female N (%)	10076 (60.5)	3337 (63.6)	3355 (57.6)	3384 (61.7)
Age, Median (IQR)	16650 (100)	13 (6–27)	12 (6–28)	13 (6–30)
Age group N (% of district)				
6 months–5 years	3893 (23.4)	1230 (23.1)	1455 (25.0)	1208 (22.0)
6–15 years	5432 (32.6)	1648 (30.9)	1962 (33.7)	1822 (33.2)
16 years and older	7325 (44.0)	2455 (46.0)	2413 (41.3)	2457 (44.8)
Season N (% of district)				
Dry season	8657 (52.0)	2748 (51.5)	3033 (52.0)	2876 (52.4)
Rainy season	7993 (48.0)	2585 (48.5)	2797 (48.0)	2611(47.6)
SES quartile^a^ N (% of district)				
Lowest	3881 (23.6)	174 (3.3)	1983 (34.5)	1724 (31.9)
Low	4015 (24.4)	666 (12.6)	1847 (32.1)	1502 (27.8)
Medium	4257 (25.9)	1321 (25.0)	1472 (25.6)	1464 (27.1)
High	4298 (26.1)	3123 (59.1)	455 (7.9)	720 (13.3)

IQR: interquartile range

^a^ SES missing 199 observations

### Distribution of microscopic and submicroscopic *P. falciparum* infection and fever

Of the 16,650 participants, 1517 (9%) had submicroscopic *P. falciparum* infections, 1570 (10%) had microscopic infection and 13,563 (81%) had no parasitaemia (Table 2). The limit of detection of PCR and microscopy in this study was ~ 2.7 parasites per microliter and 50–100 parasites per microliter, respectively [23, 25]. When comparing prevalence of infection between districts, Chikhwawa had the highest prevalence of submicroscopic (13%) and microscopic infection (17%). Participants with microscopic parasitaemia had lower median age (10 years) compared to those with submicroscopic infection (14 years) and no parasitaemia (13 years). Children between the age of 6 years to 15 years had high prevalence of submicroscopic (11.6%) and microscopic infection (15.4%) compared to other age groups. Higher prevalence of submicroscopic infections was observed in the rainy season compared to the dry season, and in the lowest to medium wealth quartiles compared to participants in the highest wealth quartile Prevalence of any parasitaemia (microscopic and submicroscopic) was highest in the lowest SES quartile (Table 2).

**Table 2:** Distribution of microscopic and submicroscopic parasitaemia and fever

Characteristic	Microscopic infections	Submicroscopic infections^a^	No parasitaemia	Fever^b^	No fever
Total	1570	1517	13563	1500	15150
District, N (row %)					
Blantyre	193 (3.6)	309 (5.8)	4831 (90.6)	361 (6.8)	4972 (93.2)
Chikhwawa	966 (17.6)	789 (13.5)	4075 (69.9)	652 (11.2)	5178 (88.8)
Thyolo	411 (7.5)	419 (7.6)	4657 (84.8)	487 (8.9)	5000 (9.1)
Age, Median (IQR)	10 (15–25)	14 (8–26)	13 (6–29)	12 (4–30)	13 (6–28)
Age group, N (row %)					
6 months–5 years	357 (9.2)	216 (6.5)	3320 (85.3)	492 (12.6)	3401 (87.4)
6–15 years	836 (15.4)	630 (11.6)	3966 (73.0)	362 (6.7)	5070 (93.3)
16 year and older	377 (5.1)	671 (9.2)	6277 (85.7)	646 (8.8)	6679 (91.2)
SES quartile, N (row %)					
Lowest	524 (13.5)	443 (11.4)	2914 (75.1)	420(10.8)	3461 (89.2)
Low	459 (11.4)	397 (9.9)	3159 (78.7)	381 (9.5)	3634 (90.5)
Medium	399 (9.4)	389 (9.1)	3469 (81.5)	366 (8.6)	3891 (91.4)
High	161 (3.8)	270 (6.3)	3867 (89.9)	308 (7.2)	3990 (92.8)
Season, N (row %)					
Rainy	1001 (2.5)	844 (10.6)	6148 (76.9)	863 (10.8)	7130 (89.2)
Dry	569 (6.6)	673 (7.8)	7415 (85.7)	637 (7.4)	8020 (92.6)

IQR: interquartile range

^a^Submicroscopic infection was defined as a positive result by real-time PCR targeting *P. falciparum* lactate dehydrogenase gene and a negative malaria microscopy result

^b^Fever defined as at least one of these three measures: reported fever in the past 2 weeks, reported fever in the past 48 h and temperature ≥ 37.5 °C measured at the time of the interview and specimen collection

Overall, 1500 of 16,650 (9%) participants had recent or concurrent fever by objective measurement or self-report. Fever was most common among residents of Chikhwawa District (11.2%), young children aged 6 months to 5 years (12.6%), people in the lowest SES quartile (10.8%), and rainy season participants (10.8%) (Table 2).

### Unadjusted analysis

The prevalence of fever among individuals with submicroscopic *P. falciparum* infection was compared to that of individuals with no parasitaemia within strata of district, age group, and season. A total of 14,239 participants were included in these final analyses after excluding participants with *P. falciparum* parasites seen on microscopy, those with missing PCR results, those who reported taking anti-malarial medications within the previous 2 weeks and those missing results for fever. Of these participants, 1408 (10%) had submicroscopic *P. falciparum* infections and 1032 (7%) had fever by objective measurement or self-report. Submicroscopic *P. falciparum* infection was significantly associated with a 49% decrease in odds of fever in the dry season (odds ratio [OR] = 0.51; 95% CI 0.33–0.78), and a non-significant increase in the odds of fever in the rainy season (OR = 1.22; 95% CI 0.94–1.58).

There was no significant association between submicroscopic *P. falciparum* infection and fever within strata of either district or age groups. Only 1.2% of the population had fever when defined by objective measure of body temperature ≥ 37.5 °C at the time of survey. In the sensitivity analysis limited to objectively measured fever, the association between submicroscopic infection and objective fever was not statistically significant within strata of either season, district or age group, though the directions of the association were generally consistent with those of the primary analysis (Table 3).

**Table 3:** Unadjusted odds ratios of the association between submicroscopic *P. falciparum* infection and fever within strata of covariates

Characteristic	Proportion with fever^a^ (%)	OR (95% CI^b^)	Proportion with objective fever^c^	OR (95% CI)
Season				
Rainy				
Submicroscopic	73/756 (9.7)	1.22 (0.94–1.58)	11/751 (1.5)	1.30 (0.68–2.47)
No parasitaemia	458/5683 (8.1)	Ref	64/5648 (1.1)	Ref
Dry				
Submicroscopic	23/652 (3.5)	0.51 (0.33–0.78)	5/652 (0.8)	0.65 (0.26–1.60)
No parasitaemia	478/7148 (6.7)	Ref	84/7115 (1.2)	Ref
District				
Blantyre				
Submicroscopic	15/282 (5.3)	0.98 (0.56–1.69)	1/282 (0.4)	0.38 (0.05–2.76)
No parasitaemia	261/4637 (5.6)	Ref	43/4615 (0.9)	Ref
Chikhwawa				
Submicroscopic	61/727 (8.4)	0.94 (0.69–1.27)	12/725 (1.7)	0.97 (0.5–1.80)
No parasitaemia	328/3762 (8.7)	Ref	64/3746 (1.7)	Ref
Thyolo				
Submicroscopic	20/399 (5.0)	0.62 (0.39–1.0)	3/396 (0.8)	0.81 (0.25–2.63)
No parasitaemia	347/4432 (7.8)	Ref	41/4402 (0.9)	Ref
Age groups				
6 months–5 years				
Submicroscopic	19/181 (10.5)	1.15 (0.70–1.88)	1/181 (0.55)	0.40 (0.06–2.97)
No parasitaemia	281/3040 (9.2)	Ref	41/3040(1.3)	Ref
6–15 years				
Submicroscopic	26/597 (4.4)	0.86 (0.56–1.31)	4/596 (0.6)	0.50 (0.81–1.40)
No parasitaemia	191/3796 (5.0)	Ref	50/3776 (1.3)	Ref
16 years & above				
Submicroscopic	51/630 (8.1)	1.05 (0.78–1.42)	11/628 (1.8)	1.85 (0.96–3.54)
No parasitaemia	464/5995 (7.7)	Ref	57/5957 (0.9)	Ref

OR: odds ratio

^a^Fever: At least one of these 3: reported fever in the past 2 weeks, reported fever in the past 48 h and temperature measured at the time of the interview and specimen collection

^b^95% CI: 95% confidence interval from mixed effect logistic regression adjusted for clustering within household and EA

^c^Objective fever: Measured temperature ≥ 37.5° (14, 166 participants)

### Final model

The final model was stratified by season and included random errors to account for clustering by household and EA. After adjusting for age and district, those with submicroscopic *P. falciparum* infections had lower odds of any fever than uninfected people in the dry season (OR = 0.52; 95% CI 0.33–0.82); no statistically significant association was observed in the rainy season (OR = 1.20; 95% CI 0.91–1.59). In the sensitivity analysis limiting the definition of fever to objective measurement of axillary temperature ≥ 37.5 °C at the time of the assessment, the estimated ORs were similar but not statistically significant in either season (Table 4).

**Table 4:** Adjusted odds ratio of the association between submicroscopic *P. falciparum* infection and fever stratified by season

Season	Fever^a^		Objective fever^b^	
	Fever/No fever	OR^c^ (95% CI)	Fever/No fever	OR (95% CI)
Rainy				
Submicroscopic	73/683	1.20 (0.91–1.59)	11/740	1.12 (0.58–2.16)
No parasitaemia	458/5225	1.00 (Ref)	64/5584	1.00 (Ref)
Dry				
Submicroscopic	23/629	0.52 (0.33–0.82)	5/647	0.55 (0.22–1.38)
No parasitaemia	478/6670	1.00 (Ref)	84/7031	1.00 (Ref)

Mixed effect logistic regression adjusted for clustering within household and EA, age and district

OR: odds ratio

95% CI: 95% confidence interval

^a^Fever: At least one of these 3: reported fever in the past 2 weeks, reported fever in the past 48 h and temperature ≥ 37.5 °C measured at the time of the interview and specimen collection

^b^Objective fever: defined as axillary temperature of ≥ 37.5 °C

## Discussion

In this analysis of results from a large, repeated cross-sectional study from southern Malawi, people with submicroscopic *P. falciparum* infection did not have higher prevalence of fever compared to uninfected people. In fact, submicroscopic infection was associated with significantly reduced odds of fever in the dry season. This study is the first large cross-sectional study that has studied the association between submicroscopic *P. falciparum* infections and fever across all age groups and seasons. This study has the advantage of exploring the relationship between fever and submicroscopic infection in a large sample size, in the rainy and dry season and across different ages and transmission settings.

While the results in the rainy season confirm findings from another cross-sectional study of Rwandan school-going children (6–10 years old) [20], they differ from a recent report from Uganda of a prospective cohort study of children and adults, which reported that children aged 2–10 years old with submicroscopic *P. falciparum* infections had increased risk of fever compared to those with no parasitaemia [19]. That study used loop-mediated isothermal amplification to detect submicroscopic infection; our PCR-based approach is slightly more sensitive [26, 27]. Importantly, the Uganda study was facility-based which may have selected for inclusion of participants when they had febrile illness episodes, compared to our community-based surveillance. In addition, the prevalence of submicroscopic infection was higher in the study population in Uganda. These are likely to be the key factors leading to our differing results.

The observation of reduced odds of fever in participants with submicroscopic malaria in the dry season was unexpected, though it has been reported previously [28]. It is possible that this was a spurious finding, but one possibility is that individuals with a history of fever would have been treated for malaria prior to the time of sample collection despite having reported no recent anti-malarial use. If this occurs in the rainy season, individuals will likely be re-infected while in the dry season when new infections are unlikely to occur. Another possibility is that chronic low-level parasitaemia has an impact on the host immune response and suppresses the febrile responses to other infections that occur [29].

Because this study was cross-sectional, the temporal relationship between submicroscopic *P. falciparum* infection and fever cannot be established, thus limiting the conclusions that can be drawn about the role of these infections in causing clinical malaria over time. History of fever was included as one of the definitions of the outcome and this may have introduced poor recall of fever episodes by participants. However, this would not bias the findings as the inaccuracies in the reporting of fever was similar in participants with submicroscopic infections and participants without infection. In this study, it was impossible to identify alternative sources of fever in participants. Assuming the distribution of non-malaria febrile illness was equally distributed among those with and without submicroscopic *P. falciparum* infection, this should not have led to a bias in the results.

Submicroscopic *P. falciparum* infections are common in many malaria endemic populations [4–6]. Findings from this study have shown that people with submicroscopic *P. falciparum* infection did not have higher prevalence of fever compared to uninfected people. However, these submicroscopic infections may persist for many months in human host and harbour transmission stages (gametocytes) of the malaria parasite [4, 23], and thus may act as transmission reservoirs. Although progress has been made in reducing malaria burden in many geographical regions, malaria elimination efforts should be designed to target all individuals with malaria infection including those with submicroscopic and asymptomatic infections. Therefore, use of molecular diagnostic methods to detect all infections will be important for successful application of interventions to interrupt transmission of infection.

## Conclusion

Submicroscopic *P. falciparum* infections are common in most malaria endemic areas but the utility of more sensitive diagnostics to identify individuals with clinical malaria disease has not been definitively established. This study demonstrates that across all age groups and a range of transmission settings, individuals with submicroscopic *P. falciparum* infections do not have higher prevalence of fever compared to those without infection. Molecular diagnostic tests should not replace microscopy and RDTs for clinical diagnosis of malaria or for treatments aimed to reduce clinical malaria. However, molecular methods remain important for identifying reservoirs of *P. falciparum* transmission.

## Data Availability

The datasets are available from the corresponding author upon appropriate request and approval from the University of Malawi College of Medicine Research and Ethics Committee.

## Abbreviations

CI: Confidence interval
EA: Enumeration area
IQR: Interquartile range
OR: Odds ratio
PCR: Polymerase chain reaction
RDT: Rapid diagnostic test
SES: Socioeconomic status
